# Smear-positive pulmonary tuberculosis and AFB examination practices according to the standard checklist of WHO’s tuberculosis laboratory assessment tool in three governmental hospitals, Eastern Ethiopia

**DOI:** 10.1186/1756-0500-7-295

**Published:** 2014-05-13

**Authors:** Abiyu Mekonnen

**Affiliations:** 1 Department of Medical Laboratory Sciences, Haramaya University, College of Health and Medical Sciences, P.O. BOX: 1154, Harar, Ethiopia

**Keywords:** Smear positive pulmonary tuberculosis, AFB staining practices, Eastern Ethiopia

## Abstract

**Background:**

Using the Directly Observed Treatment-Short course (DOTS) program the World Health Organization’s global target was to detect 70% of new sputum-smear positive PTB cases. Smear positive PTB cases are more infectious than the smear negative cases. The TB case detection rate remains very low in Ethiopia, but there are increases in smear-negative PTB diagnosis which could be attributed to several factors including poor quality of sputum smear-microscopy.

**Methods:**

A five years retrospective record review of data between September, 2007 and August, 2012 and an in-depth assessment of AFB staining practices of sputum smear using a standard checklist were made. The proportion of smear positive cases relative to overall Acid Fast Bacilli (AFB) screened was determined over a five year period to indicate the overall prevalence and the trend. Odds ratio with 95 percent confidence interval was calculated for categorical variables using multivariate Logistic Regression model to assess the strength of association.

**Result:**

A total of 1266 individuals’ data were reviewed. The majority of the study participants were male, 704 (55.6%), and rural residents, 690 (54.5%). The overall prevalence rate of smear positive PTB was 21.6%. Age categories between 15–24 and 25–34 years were independent predictors of smear positive PTB with adjusted odds ratio of 2.246 [95% CI (1.098-4.597)] and 2.267 [95% CI (1.107-4.642)], respectively. More males were affected by PTB than females with an adjusted odds ratio of 1.426 [95% CI (1.083-1.879)]. An in-depth interview with the respective laboratory chiefs showed that quality control measures for sputum smear microscopy were used at different levels of the testing activities; however, equipment function verification as a quality control measure was not accomplished regularly in all of the study hospital laboratories.

**Conclusion:**

The smear positive PTB case detection rate indicated in this study is significantly lower than the countries which met the 70% target of the World Health Organization. Lack of feedback mechanisms in the External Quality Assurance schemes of sputum smear microscopy render the opportunity for improvement difficult; Serial sputum examination showed a considerable rate of positivity in the second sputum sample when compared with the others.

## Background

The TB epidemic is in a steady, although modest and slow, decline. Nonetheless, more than nine million people still develop active TB each year and nearly two million die [1]. The Directly Observed Treatment-Short course (DOTS) strategy of tuberculosis is the World Health Organizations (WHO) recommended approach, which involves passive detection of PTB cases, primarily using sputum smear microscopy. The global target using the DOTS program was to detect 70% of new sputum-smear positive PTB cases [2]. However, only 32% of the new smear positive PTB cases estimated were detected throughout the globe [3].

Ethiopia ranked seventh among the world 22 highest burden countries, with a smear-positive TB case notification rate of 57 per 100,000 populations [4] and 54 deaths per 100,000 populations [5]. In 2010, it was reported that DOTS coverage reached 100% and TB treatment was integrated into general health services in Ethiopia. However, TB case detection rate remains very low in Ethiopia (36%) [4]. The increases in smear-negative PTB diagnosis in Ethiopia could be attributed to several factors: poor quality of sputum smear-microscopy, non adherence to diagnostic algorithm, and HIV-TB co-infection [6,7]. The magnitude of smear positive PTB in the eastern part of Ethiopia is not well studied.

Microscopy remains the mainstay of rapid TB case detection, especially for those patients who are most infectious to others, where the bacterial load involved often reflects the extent of the disease and the need for immediate treatment. The sensitivity of the direct Ziehl-Neelsen (ZN) smear depends on the diligence of the technician and on the use of appropriate technique [7,8].

External Quality Assurance (EQA) programs are needed to ensure that smears are performed and interpreted correctly and that all microscopy centres achieve an acceptable level of performance. Effective EQA programs are, however, labour-intensive and complex, requiring dedicated staffs for onsite supervisory visits and to recheck results for relatively large numbers of smears [9,10]. In addition to that, quality of AFB sputum smears microscopy just like other laboratory tests in hospital laboratories from the eastern part of Ethiopia would be compromised. This is mainly due to being a resource poor setting [11].

Direct microscopy of sputum-smear using the Ziehl-Neelsen technique is the only means to diagnose PTB in Ethiopia [12]. Assessing the AFB laboratory staining practices on the prevalence of smear positive PTB was found to be very crucial. In Ethiopia, this inquiry has not been adequately addressed, and no studies have previously been conducted in the eastern part of the country. Therefore, this study was designed to assess the magnitude of smear positive PTB. Further the study looked at AFB Staining practice of sputum smear in the hospital laboratories. Understanding the prevalence of smear positive PTB in the eastern part of the country could provide data relevant for TB programmers, researchers, decision makers and stakeholders striving to the prevention and control of TB.

## Methods

### Study setting and period

**Figure 1:**
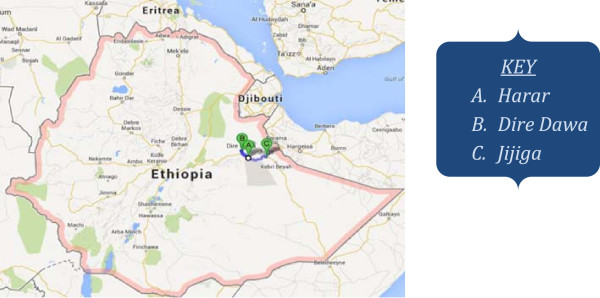
**Map indicating study areas: Harar, Dire Dawa, and Jijiga, Eastern Ethiopia. ** Source: http://www.goodtogrowinc.org/where-we-work.html.

The study was conducted amongst hospitals in Eastern Ethiopia. It includes Dire Dawa, DilChora hospital; Harar, HiwotFana hospital and Jijiga, Karamara hospital. Dire Dawa administration is a city located 500 km away from the capital Addis Ababa. Harar is a town located 510 km away from Addis Ababa and is the capital of the Harari regional state. Jijiga is a town located about 615 km away from Addis Ababa, which is the capital of the Somali region. The hospitals were initially conveniently selected for this study for budgetary reason. The hospitals were also assumed to represent two regional states and an administration in the eastern part of Ethiopia. HiwotFana hospital was one of the two public hospitals in Harar town,and included by lottery method; whereasKaramara and DilChora hospitals were the only public hospitals in Jijiga and Dire Dawa, respectively. The three hospitals were estimated to serving for 1–1.5 million people each. Data was collected from September to October, 2012 (Figure 1).

### Study design

From September, 2007 to August, 2012, a cross-sectional retrospective study based on record review, was conducted to determine the trend and prevalence of smear positive PTB. This was supplemented qualitatively by in-depth interview with the respective hospital laboratory chiefs. The interview explored the laboratories overall practices of AFB examination in the last five years.

### Study population and samples

In the three hospitals in the last five years there were 8862 patients screened for the AFB. The study population includes individuals screened for AFB in the study hospitals. In order to determine the prevalence the following sample size calculation was used. The population proportion taken was 50% as there was no previous study in the setting, marginal error considered was 4% with a design effect of 2, and the confidence level used was 95% [13]. And with the added 10% non response rate the final sample size was 1320. The sample size obtained during the study was 1266, and every 7^th^ of the total 8862 patients were included to reach this sample size. Four hundred twenty-two individuals were selected from each of the three hospitals.

Therefore, a total of 1266 individuals with a complete data register including age, sex, residence and results of serial sputum smear examinations were included. However, those with incomplete information for any of the above variables were excluded from study.

In-depth interview using WHO’s Tuberculosis Laboratory Assessment tool were conducted with chiefs of the respective hospital laboratories to qualitatively supplement the secondary data. The informant chiefs were serving in the hospital laboratories for more than five years during the data collection period and were recognized as knowledgeable on each and every activity of the respective laboratories.

### Data collection and quality control

For the secondary data a standard questionnaire was used for recording information extracted from the TB registry. Data about gender, age, residence and serial sputum examination results of patients were obtained from the registry. Supplementary qualitative information about AFB staining practices were explored by in-depth interview using WHO’s Tuberculosis Laboratory Assessment tool (3^rd^ draft, 2002) with chiefs of the respective hospital labs. Smear positive PTB cases were defined as those individuals with two or more sputum smears positive for AFB examination. Data was collected by three trained laboratory technologists who were working at Haramaya University under intensive supervision of the researcher. The data collectors had two days training which focused on how to collect complete data from AFB record books and how to conduct in-depth interviews with the hospital laboratory chiefs using the standard checklist provided. All completed data collection forms were examined for clarity and consistency by the principal investigator of the study.

### Data analysis

The data were entered into SPSS version-16, and Chi-square and P value computation was accomplished to depict the possible association that might exist between the dependent and independent variables. Odds ratio with 95 percent confidence interval was calculated for categorical variables using multivariate Logistic Regression model to assess the strength of association. The dependent variable of this study was the magnitude of smear positive PTB, whereas the independent variables were socio-demographic characteristic of the study participants and the study health service institutions.

### Ethical consideration

The study was ethically approved by Haramaya University Institutional Research and Ethical Review Committee, and the data collection was carried out after submitting the ethical clearance letter to the respective hospital administrations and chiefs of laboratories, and informed consent were given by chiefs of the hospital laboratories.

## Results

### Characteristics of study participants

A total of 1266 individuals, with a response rate of 95.9%, 422 from each hospital (HiwotFana, DilChora, and Karamara), were studied. The majority of the study participants were male, 704 (55.6%), and rural residents, 690 (54.5%). The median age of the study population was 30 years with a standard deviation of 14.9 years (Table 1).

**Table 1:** Characteristic of study participants by study hospitals in three governmental hospitals, Eastern Ethiopia, 2007-2012

**Characteristic**	**Karamara hospital no. (%)**	**DilChora hospital no. (%)**	**HiwotFana hospital no. (%)**	**Total**
**Gender**				
Female	180 (42.7)	178 (42.2)	204 (48.3)	562 (44.4)
Male	242 (57.3)	244 (57.8)	218 (51.7)	704 (55.6)
Total	422 (33.3)	422 (33.3)	422 (33.3)	1266 (100)
**Age group (in years)**				
<15	35 (8.3)	4 (0.9)	36 (8.5)	75 (5.9)
15-24	130 (30.8)	118 (28)	116 (27.5)	364 (28.8)
25-34	100 (23.7)	124 (30.1)	134 (31.8)	358 (28.3)
35-44	65 (15.4)	66 (15.6)	73 (17.3)	204 (16.1)
45-55	51 (12.1)	63 (14.9)	35 (8.3)	149 (11.8)
>55	41 (9.7)	47 (11.1)	28 (6.6)	116 (9.2)
Total	422 (33.3)	422 (33.3)	422 (33.3)	1266 (100)
**Residence**				
Rural	253 (60)	229 (54.3)	208 (49.3)	690 (54.5)
Urban	169 (40)	193 (45.7)	214 (50.7)	576 (45.5)
Total	422 (33.3)	422 (33.3)	422 (33.3)	1266 (100)

The chiefs of the three study hospital laboratories interviewed were male between the age of 33 and 37; who had been serving in the respective laboratories for more than five years.

The data obtained from the three study hospital laboratories showed notable similarities in both contents and record keeping practice. Despite some incompleteness of information in the AFB records, the differences in record keeping between the study hospital laboratories were not significant.

### Smear Positive Pulmonary Tuberculosis

The overall prevalence rate of smear positive PTB was 21.6%. Almost 25% of the males and 19% of the females studied were positive for sputum smear microscopy. Smear positive PTB was significantly higher in rural than urban residents [AOR: 1.573, 95% CI (1.193-2.076)] (Table 2).

**Table 2:** Socio demographic characteristic of the study participants and its association with smear positive pulmonary TB in three governmental hospitals, Eastern Ethiopia, 2007-2012

**Characteristic**	**Total examined no. (%)**	**Smear positive no. (%)**^**¥**^	**COR (95% CI)**	**AOR (95% CI)**
**Gender**				
Female	562 (44.4)	108 (19.2)	1	1
Male	704 (55.6)	175 (24.9)	1.391 (1.061-1.823)*	1.426 (1.083-1.879)*
**Age group (in years)**				
<15	75 (5.9)	10 (13.3)	1	1
15-24	364 (28.7)	92 (25.3)	2.199 (1.085-4.456)*	2.246 (1.098-4.597)*
25-34	358 (28.3)	93 (26.0)	2.281 (1.126-4.623)*	2.267 (1.107-4.642)*
35-44	204 (16.1)	46 (22.5)	1.892 (0.901-3.976)	1.858 (0.875-3.943)
45-55	149 (11.8)	24 (16.1)	1.248 (0.563-2.767)	1.280 (0.569-2.878)
>55	116 (9.2)	18 (15.5)	1.194 (0.518-2.749)	1.201 (0.514-2.804)
**Residence**				
Rural	690 (54.5)	177 (25.7)	1.530 (1.166-2.007)**	1.573 (1.193-2.076)**
Urban	576 (45.5)	106 (18.4)	1	1
**Study hospital**				
Karamara	422 (33.33)	89 (21.1)	1	1
DilChora	422 (33.33)	80 (18.9)	0.875 (0.624-1.227)	0.871 (0.617-1.228)
HiwotFana	422 (33.33)	114 (27)	1.385 (1.008-1.903)*	1.449 (1.047-2.006)*

COR: Crude Odds Ratio; AOR: Adjusted Odds Ratio; CI: Confidence Interval.

*P < 0.05; **: p < 0.01.

^¥^the percentage is calculated from the total examined in the respective characteristic.

Age categories between 15–24 and 25–34 years were independent predictors of smear positive PTB with adjusted odds ratio of 2.246 [95% CI (1.098-4.597)] and 2.267 [95% CI (1.107-4.642)], respectively. More males were affected by PTB than females with an adjusted odds ratio of 1.426 [95% CI (1.083-1.879)] (Table 2).

Smear positive pulmonary TB was highly prevalent at HiwotFana hospital followed by Karamara and DilChora hospitals with a prevalence of 27%, 21.1%, and 18.9%, respectively. At the same time, it was significantly higher at HiwotFana hospital [AOR: 1.449, 95% CI (1.047-2.006)] (Table 2).

**Figure 2:**
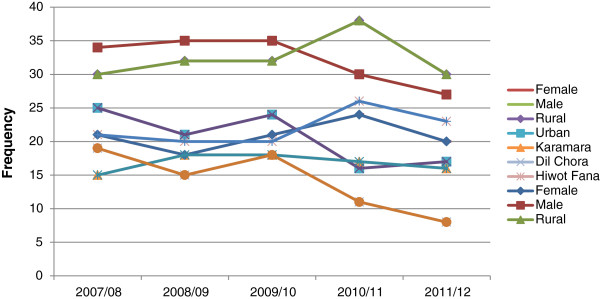
Five years trend of smear positive PTB by gender, residence and study hospital, Eastern Ethiopia, 2007–2012.

The trend of smear positive PTB does not show any considerable change during the five years of examination. The prevalence of smear positive PTB was higher in males than their female counterparts and also in rural than their urban counterparts (Figure 2).

Out of the 1266 study participants, 244 (19.3%), 33 (2.6%) and 13 (1.0%) were with three positive smears, two positive smears and one positive smear, respectively. However, the remaining 976 (77.1%) were negative for AFB examination (Table 3).

**Table 3:** Serial sputum sample results by year of examination, Eastern Ethiopia

**Serial sputum sample results**	**Year of examination**					**Total**
	**2007/08 no. (%)**	**2008/09 no. (%)**	**2009/10 no. (%)**	**2010/11 no. (%)**	**2011/12 no. (%)**	
Three positive smears	53 (21.0)	48 (18.8)	52 (20.6)	49 (19.2)	42 (16.7)	244 (19.3)
Two positive smears	2 (0.8)	9 (3.5)	7 (2.7)	10 (3.9)	5 (2.0)	33 (2.6)
One positive smear	1 (0.4)	3 (1.2)	2 (0.8)	0 (0)	7 (2.8)	13 (1.0)
Negative smears	196 (77.8)	195 (76.5)	191 (75.6)	196 (76.9)	198 (78.6)	976 (77.1)
Total	252 (19.9)	255 (20.1)	252 (19.9)	255 (20.1)	252 (19.9)	1266 (100)

No statistically significant variability was observed between the first spot samples, second morning samples and the third spot samples by year of examinations. In this study, the rate of positivity was increased from 21.6% in the first spot sample to 22.4% in the second morning sample. However, no increment was observed from the third spot sample, rather there was a reduction in the positivity rate to 20.9% (Table 4).

**Table 4:** frequency of smear positive serial sputum samples by year of examination, Eastern Ethiopia

**Type of sputum sample**	**Year of examination**					**Total**	**P-value**
	**2007/08 no. (%)**	**2008/09 no. (%)**	**2009/10 no. (%)**	**2010/11 no. (%)**	**2011/12 no. (%)**		
**First spot sample**							P = 0.98
Positive	56 (22.2)	55 (21.6)	56 (22.2)	55 (21.6)	51 (20.2)	273 (21.6)	
Negative	196 (77.8)	200 (78.4)	196 (77.8)	200 (78.4)	201 (79.8)	993 (78.4)	
Total	252 (19.9)	255 (20.1)	252 (19.9)	255 (20.1)	252 (19.9)	1266 (100)	
**Second morning sputum sample**							P = 0.91
Positive	57 (22.6)	58 (22.7)	60 (23.8)	58 (22.7)	50 (19.8)	283 (22.4)	
Negative	195 (77.4)	197 (77.3)	192 (76.2)	197 (77.3)	202 (80.2)	983 (77.6)	
Total	252 (19.9)	255 (20.1)	252 (19.9)	255 (20.1)	252 (19.9)	1266 (100)	
**Third spot sample**							P = 0.89
Positive	55 (21.8)	53 (20.8)	56 (22.2)	54 (21.2)	47 (18.7)	265 (20.9)	
Negative	197 (78.2)	202 (79.2)	196 (77.8)	201 (78.8)	205 (81.3)	1001 (79.1)	
Total	252 (19.9)	255 (20.1)	252 (19.9)	255 (20.1)	252 (19.9)	1266 (100)	

Serial sputum examinations do not show any significant variability between the first spot samples, second morning sputum samples and the third spot samples by year of examinations. On average 4.5 suspected cases had to be screened to diagnose one smear positive case. A total of 283 (22.4%) individuals with suspected PTB had at least one positive smear. Of these, 273 (21.6%) were positive from the first specimen; the other 10 (0.8%) were positive on the second morning specimen but not the first. However, eight and eighteen specimens were negative from the first spot specimen and the second morning specimen to the third specimen, respectively (Table 4).

### AFB examination practices according to the standard checklist of WHO’s TB lab assessment tool

An in-depth interview with the chief of each hospital laboratory was conducted using a standard checklist of WHO’s Tuberculosis Laboratory Assessment tool (3^rd^ draft, 2002). The interview assessed the laboratories overall practices in the five year period. The assessment showed considerable similarity between the study hospital laboratories practice, with the exception of some minor inconsistencies (Table 5).

**Table 5:** AFB examination practices according to the standard checklist of WHO’s TB lab assessment tool, Eastern Ethiopia, 2007-2012

**Criteria**	**Yes (no. %)**	**No (no. %)**
**Presence of national TB lab manual**	0 (0)	3 (100)
**Presence of national guideline (or protocol) of Quality Assurance**	0 (0)	3 (100)
**Quality control measures during**		
Specimen reception/handling	2 (66.7)	1 (33.3)
Stains preparation	3 (100)	0 (0)
Staining	1 (33.3)	2 (66.7)
Equipment function	1 (33.3)	2 (66.7)
Reading and reporting	3 (100)	0 (0)
**Presence of Internal quality assessment program (IQA)**	3 (100)	0 (0)
**Participation in External Quality Assessment program (EQA)**	3 (100)	0 (0)
**Presence of feedback mechanism on results of EQA**	0 (0)	3 (100)
**Trainings on safe lab practices (safety)**	2 (66.7)	1 (33.3)
**Regular health check-up of lab workers**	0 (0)	3 (100)
**Availability of in-service trainings on sputum smear microscopy**	2 (66.7)	1 (33.3)
**Frequent interruption of lab work due to shortage of supplies for sputum smear microscopy**	1 (33.3)	2 (66.7)
**Presence and adequacy of maintenance system for equipments**	0 (0)	3 (66.7)

All three laboratories had no problems with the number and qualification of lab staffs to perform sputum smear microscopy. However, to the best of their knowledge, the chiefs indicated the absence of a national TB manual and guideline or protocol of quality assurance of smear microscopy, either in the respective hospital laboratories or in the country.

Quality control measures for sputum smear microscopy were used at different levels of the testing activities; quality control during specimen reception/handling by using clearly described specimen rejection criteria were used in one of the hospitals studied, whereas, quality control during stain preparation and in the staining procedure along with quality control measures on reading and reporting of results was performed with some irregularity in the study hospitals. However, equipment function verification as a quality control measure was not accomplished regularly in all of the study hospital labs.

All the study hospital laboratories regularly apply internal quality assurance schemes for sputum smear microscopy at the beginning of every month. Even though there are no regular feedback mechanisms to detect areas that need improvement and no means to ensure the implementation of any corrective actions recommended, all the study hospital labs were involved in an external quality assurance program organized by the respective regional laboratories using slide rechecking methods.

The work load of the lab staffs on sputum smear microscopy was about 7 to 9 smears examined per worker per day. The lab staffs implementation of safety practice in sputum smear microscopy appeared to be of good quality. They used appropriate disinfectants regularly and disposed of waste based on biosafety requirements. However, availability of training on safe lab practices was scarce, and there were no regular health check-ups of laboratory workers in any of the study hospitals using chest x-ray, sputum examination or any other mechanisms.

The role of in-service training to enhance quality laboratory service is highly significant. Some of the laboratory workers at Karamara hospital did not receive in-service training on sputum smear microscopy, whereas those in DilChora and HiwotFana hospitals attended training organized by Haramaya University in collaboration with Ethiopian Health and Nutrition Research Institute (EHNRI) and Centers for Disease Control and Prevention- Ethiopia (CDC-E) at different training periods.

The procurement system for lab supplies including reagents, consumables and equipment functioned efficiently in the sputum smear microscopy as compared with other supplies required in clinical laboratory services. This was indicated by the very rare interruption of the test due to shortage of supplies. However, the maintenance system for equipment in all the study hospitals was highly deficient resulting in microscopes and other equipment being deposited in storage rooms when they may only have required minor repairs.

## Discussion

Smear positive PTB was highly prevalent between the ages of 15–34 years, and more males were affected than females. Unlike other studies which have shown a higher prevalence of PTB in the elderly (>45 years) [14], this study revealed that the risk to get smear positive pulmonary TB was about a 2.3 times higher among people in the age group of 15–34 years than the other age categories. This might be explained by the more interactive nature of the people in this age category when compared with those under 15 years and the elderly. This could also be related to the higher association of PTB with HIV/AIDS [15]. The higher proportion of a smear-positive sputum sample in males than females is consistent with data from Ethiopia [2] and other countries also [16,17], and could reflect occupational, behavioral or immunological factors that contribute to risk.

Rural residents had about a two times higher risk of smear –positive PTB than that of their urban counter-parts. This could be due to lack of awareness and poor housing conditions among the rural inhabitants than the urban dwellers. A study from Tigray showed that rural residents were less knowledgeable about PTB than their urban counterparts [18]. Furthermore, differences in literacy rate and access to health services could account for the variation in TB-knowledge among urban and rural respondents [19], and for the variation in the prevalence of PTB.

This study revealed the overall prevalence of smear positive PTB during the five year period to be 21.6%, which indicates that the disease is of major clinical significance in the study areas. This finding is higher than studies from south west Ethiopia which showed a prevalence of 10.9% from Agaro [20] and 8.5% from Jimma University specialized hospital [21]. This difference could be due to variability in the awareness of communities for early diagnosis and treatment or the increased susceptibility to pulmonary TB due to excessive exposure to predisposing factors like smoking and khat chewing habits in the study areas of the east when compared to south western part of the nation [22]. Ethiopia is among the countries with a high burden of TB. Despite the implementation of control strategies against PTB in the 1960’s the impact in curbing the magnitude of the problem has been insignificant [23]. This finding again emphasizes the call for devising effective preventive and control strategies against PTB both in the eastern Ethiopia and other parts of the country.

The increase in the prevalence of smear positive TB in the study area could also be explained by the effective active follow up of the nongovernmental organizations (NGOs) like International Center for AIDS care and treatment Program- Ethiopia (ICAP-E) who focused mainly on TB-laboratory activities in the Eastern part of Ethiopia, that could enhance the quality of AFB testing procedure and hence the rate of positivity. A study from Tigray showed a comparable prevalence of smear-positive PTB to be 24.6% [6], and an increased proportion of smear positive cases were also reported from southern region of Ethiopia [24].

The principal WHO measure of case detection is an increment for new smear-positive cases in DOTS program [25]. However, this finding from eastern Ethiopia is very low when compared with the case detection rate achieved in Africa (46%), and the 77 countries which met the 70% target by the end of 2006. According to this WHO report, Ethiopia is accounting for more than one-quarter of “missing” cases [25]. This decreased smear positive case detection rate in the study area and in the country may show a lack of emphasis on sputum smear microscopy or an incomplete geographical coverage of DOTS.

The decreases in the magnitude of smear positive PTB as compared with other areas could also be explained by the cumulative effects of contributing factors including: lack of regular in-service trainings on sputum smear microscopy; absence of quality assurance guidelines or protocols of smear microscopy; and nonexistence of equipment function verification scheme as quality control measure in the study hospitals among others.

Smear positive pulmonary TB showed a statistically significant variability between the study hospitals. It was highly prevalent at HiwotFana followed by Karamara and DilChora hospitals, respectively. This difference could be attributed to; variations in geographical coverage of DOTS strategy in the communities; differences in the respective hospital administrations; differences in commitment of laboratory staffs and opportunities for training that enhances quality services in carrying out all pre analytical, analytical and post analytical procedures for sputum smear microscopy. In addition, differences in frequency of monitoring and support from regional laboratories and NGO’s like ICAP-E could contribute to this variability.

The study does have limitations. The retrospective study design limited our ability to gather data about factors that may influence quality of sputum smear microscopy from the patients’ side, for instance, quality of sputum specimens presented for examination and HIV status of patients would have been investigated. In addition, even though a detailed interview was conducted with the respective hospital laboratory chiefs to assess important factors associated with quality of sputum smear microscopy, it was difficult to judge the actual pre-analytical, analytical and post-analytical sputum smear microscopy procedures employed in the laboratories in the past five years.

## Conclusion

The overall prevalence of sputum smear positive cases of 21.6% indicates that the disease is of major clinical significance in the study areas. However, the case detection rate pointed out in this study is significantly lower than the target set by WHO; this signifies the need to improve the rigor of the sputum smear microscopy test. The most vulnerable groups were males, rural residents and individuals in the age range 15–34 years. Designing and implementing preventive measures targeting these gender; residence and age categories is recommended. An opportunity to improve the situation has been missed due to the lack of feedback mechanisms in the external quality assurance program. Serial sputum examination showed a considerable rate of positivity in the second morning sputum sample when compared with the others. However, the third spot sputum sample showed no positivity increase in comparison to the first spot and the second morning sputum samples. Further studies are also recommended.

## Competing interests

The author declares that he has no competing interests associated with the publication of this manuscript.

## Authors’ information

AM, BSc in Medical Laboratory Technology, MSc in Tropical and Infectious Diseases, is working at Haramaya University, College of Health and Medical Sciences, Department of Medical Laboratory Sciences.
